# Clinically relevant prognostic and predictive markers for immune-checkpoint-inhibitor (ICI) therapy in non-small cell lung cancer (NSCLC)

**DOI:** 10.1186/s12885-020-07690-8

**Published:** 2020-12-03

**Authors:** Wolfgang M. Brueckl, Joachim H. Ficker, Gloria Zeitler

**Affiliations:** 1 Department of Respiratory Medicine, Allergology and Sleep Medicine / Nuremberg Lung Cancer Center, Paracelsus Medical University, General Hospital Nuremberg, Prof.-Ernst-Nathan-Str. 1, 90419 Nuremberg, Germany; 2 Paracelsus Medical Private University Nuremberg, Prof.-Ernst-Nathan-Str. 1, 90419 Nuremberg, Germany

**Keywords:** Immunotherapy, Immune checkpoint inhibitor (ICI), PD-L1 inhibitor, NSCLC, Prognostic, Predictive, Biomarker

## Abstract

**Background:**

Immune checkpoint inhibitors (ICI) either alone or in combination with chemotherapy have expanded our choice of agents for the palliative treatment of non-small cell lung cancer (NSCLC) patients. Unfortunately, not all patients will experience favorable response to treatment with ICI and may even suffer from severe side effects. Therefore, prognostic and predictive markers, beyond programmed death ligand 1 (PD-L1) expression status, are of utmost importance for decision making in the palliative treatment. This review focuses on clinical, laboratory and genetic markers, most of them easily to obtain in the daily clinical practice.

**Results:**

Recently, a number of prognostic and predictive factors in association to palliative ICI therapy have been described in NSCLC. Besides biometric parameters and clinical characteristics of the tumor, there are useful markers from routine blood sampling as well as innovative soluble genetic markers which can be determined before and during ICI treatment. Additionally, the level of evidence is noted.

**Conclusions:**

These factors can be helpful to predict patients’ outcome and tumor response to ICI. They should be implemented prospectively in ICI based clinical trials to develop reliable algorithms for palliative NSCLC treatment.

## Key points

- PD-L1 alone is the only approved prognostic biomarker for ICI therapy in NSCLC patients to date.
- Patients in a poor PS status or those needing oral corticosteroids or antibiotics before treatment are probably no good candidates for an ICI based therapy unless these medications can be stopped. Additionally, patients with a malignant pleural effusion might not have a benefit from immunotherapy.
- In addition to clinical features, laboratory parameters like CRP or the neutrophil-to-lymphocyte ration (NLR) or genetic factors like sPD-L1 or ctDNA in the blood measured before and during ICI treatment may become evident as prognostic or predictive markers in the near future. As there is a difference in predictive value between TMB, KRAS and STK11/KEAP1 between trial with ICI monotherapy and those with ICI-combinations those markers are not yet ready for being used in the clinical setting.

## Background

In recent years the choice of standard therapeutic options for metastatic NSCLC patients has been expanded by immune-checkpoint-inhibitor (ICI) therapy either as monotherapy or in combination with chemotherapy, anti-angiogenic antibodies or with other forms of ICI. ICI, namely antibodies directed against PD-1 or PD-L1, were approved for the 2nd or 3rd line treatment of metastatic NSCLC in patients without treatable driver mutations in 2015 [1–7]. Since then, ICIs have been approved in the 1st line setting, either alone in tumors with PD-L1 ≥ 50% expression or in combination with chemotherapy independent of the receptor status [8–13]. Some patients treated with ICIs have exceptionally long-lasting responses and survival. For instance, up to 16% of NSCLC stage IV patients treated with the PD-1 inhibitor nivolumab in 2nd line and 31.9% of patients in 1st line with the PD-1 inhibitor pembrolizumab survived 5 years, respectively [11, 14, 15].

On the other hand, many mortality curves of the ICI therapy showed an increased risk of death during the initial phase of ICI treatment. Reasons for this phenomenon may be primary resistance with hyper progressive tumor growth as well as immune-related adverse events. Therefore, it is of utmost importance for the treating physician to reliably know whether the ICI will be beneficial for the individual NSCLC patient or not. This prediction of response can be based on prognostic and, if other treatment options are available, predictive factors. However, in most pivotal trials those factors are not analyzed comprehensively. Furthermore, due to strict exclusion criteria, those trials do not represent the broad spectrum of patients in the daily clinical practice presenting with e.g. low performance status and possible co-morbidities. Harvey et al. recently presented a revised and broadened list of clinical trial eligibility criteria to enable more patients to participate, make future trial populations more representative and results more generalizable [16]. They focused on including patients with other primary cancers, brain metastases irrespective of treatment status and clinical stability as well as a creatinine clearance > 30 mL/min, what enabled nearly twice as many NSCLC patients to consider trial participation in their real-world data analysis. It is of paramount importance that clinical trial sponsors adopt these criteria to increase the practical value of clinical studies.

PD-L1 expression on cancer cells determined by immunohistochemistry to date is the only fully in clinical practice implemented, FDA-approved biomarker for ICI response so far. The use of the ICI-agents pembrolizumab and atezolizumab as monotherapy in the palliative 1st line setting is therefore only approved for tumors with a PD-L1 Tumor Proportion Score (TPS-Score) of 50% or higher [10, 17, 18]. For second line in stage IV with pembrolizumab the tumor must have a PD-L1 expression of at least 1% [19–21].

Nevertheless, the predictive power of PD-L1 expression is limited:

- the expression of PD-L1 seems to be lower in primary tumor samples than in metastases. This indicates that an advanced disease may be more likely to express PD-L1 than an earlier stage disease [22].
- the extent of PD-L1 expression in metastases varies modestly by anatomic site. The proportion of cases with “PD-L1 high expression” was greatest in lymph nodes (30% PD-L1 high) and least in bone (16% PD-L1 high), so lymph node sampling may require an adjusted scale [22].
- among patients with primary tumor tissue sampling, there was lower PD-L1 expression when resection specimens were used compared to biopsy samples [22]. These weaknesses of PD-L1 emphasize the demand of additional markers.

The biomarkers discussed in this review will be categorized into prognostic and predictive markers by the definition used by Clark et al. [23, 24]. They defined a prognostic marker as a predictor of the natural history of disease. These markers are associated with the clinical outcome in the absence of therapy or when a standard therapy is applied [24, 25]. Predictive markers on the other hand indicate a differential benefit from a particular therapy. Furthermore, we divided the potential markers into A) biometric data and clinical findings, B) blood based parameters, and C) tumor based immunohistochemical and DNA markers [24, 25]. Details of the following data and the studies included in this review are summarized in Table 1A, B and C.

**Table 1:** Prognostic and predictive factors in A) clinical findings, B) laboratory values and C) immunohistochemistry and genetics for ICI treatment in NSCLC discussed in this review in more detail. Level of evidence is given for every marker. Sequence of studies as appearance in the review. n.s., not significant; + statistically significant positive prognostic/predictive factor; − statistically significant negative prognostic/predictive factor

Authors	Year	ICI	n	Biomarker	Level of evidence	PFS	OS
**Clinical findings**							
Conforti et al. [26]	2018	ICI single agent therapy	11,351	male gender	2A	NS	+
Conforti et al. [27]	2019	PD-L1/PD-1 inhibitor + chemotherapy	4923	female gender	2A	+	+
Kawachi et al. [28]	2020	pembrolizumab	213	pleural effusion	2B	–	NS
				ECOG PS < 2	2B	+	NS
Naqash et al. [29]	2020	nivolumab	531	irAE	2B	+	+
				ECOG PS < 2	2B	+	+
				ICI duration > 3 months	2B	+	+
Dall’Olio et al. [30]	2020	immunotherapy	3600	ECOG PS ≥ 2	2A	–	–
Cortellini et al. [31]	2020	PD-1/PD-L1 inhibitors	1070	BMI ≥ 25 kg/m^2	3B	+	+
Shepshelovich et al. [32]	2019	various assessments	25,439	BMI ≥ 40 / < 18,5 kg/m^2	2A	NS	–
Magri et al. [33]	2019	nivolumab	46	weight loss before treatment > 5%	2B	NS	–
Kang et al. [34]	2018	PD-1/PD-L1 inhibitors	51	pleural or pericardial metastasis	3B	–	NS
Adachi et al. [35]	2020	nivolumab	296	driver mutation positivity	3B	–	NS
				CRP ≥ 1 mg/dL	3B	–	NS
				liver metastasis	3B	–	NS
				pleural effusion	3B	–	NS
				ALI ≥ 18	3B	+	NS
				baseline steroid use	3B	–	NS
Reck et al. [36]	2019	ABCP	1202	liver metastasis	1A	+	+
Sridhar et al. [37]	2019	Durvalumab	569	liver metastasis	1A	–	–
Scott et al. [38]	2018	nivolumab	210	baseline steroid use	3B	NS	–
Fuca et al. [39]	2019	PD-1/PD-L1 inhibitors	151	baseline steroid use	3B	–	–
Arbour et al. [40]	2018	PD-1/PD-L1 inhibitors	640	baseline steroid use	3B	–	–
Routy et al. [41]	2018	PD-1/PD-L1 inhibitors	379	antibiotic use (60 days before thx)	2B	–	–
Derosa et al. [42]	2018	PD-1/PD-L1 inhibitors	239	antibiotic use (30 days before thx)	3B	NS	–
Ahmed et al. [43]	2018	PD-1/PD-L1 inhibitors	60	broad spectrum antibiotics	2B	–	NS
Chalabi et al. [44]	2020	atezolizumab + docetaxel	1512	antibiotic use	1A	NS	–
Hakozaki et al. [45]	2020	nivolumab, pembrolizumab or atezolizumab	90	decreased *Ruminococcaceae UCG 13* and *Agathobacter*	2B	–	–
**Laboratory values**							
Cao et al. [46]	2018	nivolumab	1225	NLR ≥ 5	3A	–	–
Zhang et al. [47]	2020	ICI therapy 2nd-line	74	NLR ≥ 3	3B	NS	NS
Iivanainen et al. [48]	2019	PD-1/PD-L1 inhibitors	160	NLR > 2,65	2B	–	–
				CRP ≥ 10 mg/mL	2B	–	–
Liu et al. [49]	2019	nivolumab	44	NLR ≤ 3.07	3B	+	+
				SII ≤ 603.5	3B	+	+
				PLR ≤ 144	3B	+	+
Mezquita et al. [50]	2018	PD-1/PD-L1 inhibitors	628	positive LIPI	2B	NS	–
				LDH > UNL	2B	NS	+
Passaro et al. [51]	2020	nivolumab	53	Gr-MDSC < 6 cells/μl	2B	+	+
				NC < 5840/μl	2B	+	+
				eosinophils > 90/μl	2B	+	+
				NLR < 3	2B	+	+
Tanizaki et al. [52]	2018	nivolumab	134	ALC > 1000/μ	3B	+	NS
				AEC > 150/μL	3B	+	NS
				ANC > 7500/μL	3B	–	–
Zhang et al. [53]	2019	PD-1/PD-L1 inhibitors	1136	LDH > UNL	3A	+	+
Naqash et al. [54]	2018	nivolumab	87	CRP > 50 mg/L	2B	NS	–
				NLR > 6,5	2B	NS	–
				PNI < 31,5	2B	NS	–
**Immunohistochemistry and genetic parameters**							
Okuma et al. [55]	2018	nivolumab	39	sPD-L1 > 3357 ng/mL	2B	NS	+
Costantini et al. [56]	2018	nivolumab	43	low sPD-L1	2B	+	+
Shibaki et al. [57]	2020	nivolumab, pembrolizumab	235	low serum VEGF	3B	+	+
Wang et al. [58]	2019	PD-1/PD-L1 inhibitors	98	bTMB > 6	3B	+	NS
Rizvi et al. [59]	2020	durvalumab vs. durvalumab + tremelimumab vs. chemo	1118	bTMB ≥20 mut/mb	1B	NS	+
Paz-Ares et al. [60]	2019	pembrolizumab + chemo vs. chemo	605	tTMB	1A	NS	NS
Herbst et al. [61]	2019	pembrolizumab vs. platinum-based chemo	1274	KRAS & pembrolizumab	1B	+	+
Gadgeel et al. [62]	2019	pembrolizumab+premetrexed+platinum vs. placebo+premetrexed+platinum	616	KRAS & pembrolizumab +chemo	1B	+	+
Skoulidis et al. [63]	2018	PD-1/PD-L1 inhibitors	1208	STK11/LKB1	2B	–	–
Arbour et al. [64]	2019	ICI inhibitors vs. chemotherapy	330	KEAP1/NFE2L2	2B	NS	–
Cho et al. [65]	2020	pembrolizumab vs. platinum-based chemo	1274	STK11 or KEAP1	1B	NS	NS
Bratman et al. [66]	2020	pembrolizumab	94	low baseline ct-DNA concentration	2A	+	+
Zulato et al. [67]	2020	ICI therapy	34	new KRAS mutation becoming apparent after 3–4 weeks	2B	–	–

**Fig. 1:**
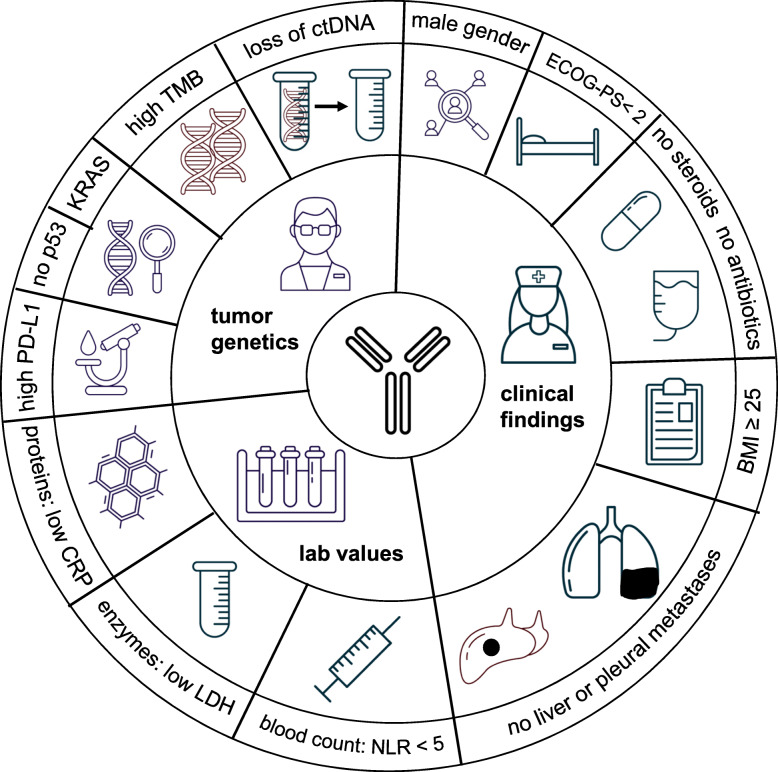
Comprehensive overview about the different positive prognostic and predictive factors in treating patients with ICI discussed in the review

Due to the growing importance of ICI in the treatment of NSCLC, a number of recent reviews have been published that focus on prognostic factors. However, most of them focused on PD-L1 and TMB as biomarkers, went into detail on specifically selected pathways, e.g. the WNT signaling pathways, or summarized prognostic factors for both treatment success and for overcoming resistance to ICI [68–70]. Beside including the latest results for biomarkers from post hoc analyses of recently published Phase III trials, this review focuses on factors that are easily identified in daily clinical practice prior to initiating ICI based therapy. In addition, we have briefly presented the scientific basis explaining why each of these biomarkers may be prognostic for the effect of ICI therapy and provide the available evidence level for the predictive value of the biomarker. Finally, we provide a comprehensive chart overview of the various positive prognostic and predictive factors in the treatment of patients with ICI discussed in this review. (Fig. 1).

The electronic databases Pubmed and PMC as well as abstracts from the ESMO, ASCO, AACR and WCLC congresses were systematically searched to identify eligible studies evaluating predictive and prognostic biomarkers in NSCLC patients receiving ICI therapy. There was no limitation of language and study type. All resources in this article were classified with the Oxford Centre for Evidence-Based Medicine 2011 Levels of Evidence table and evaluated the endpoints PFS or OS [71, 72].

## Main text

### Clinical findings predicting the efficacy of ICI therapy

#### Gender

Gender is a relevant factor that modulates immune response and has effects on the expression and function of PD-L1 and PD-1 [26, 73, 74]. Many studies on various tumor types including NSCLC could show that not women but men have an increased efficacy of single agent ICI treatment [4, 26, 75, 76]. On the other hand, women apparently have a greater advantage from a combination of ICI and chemotherapy [8, 12, 21, 27, 60]. A possible hypothesis to explain this finding could be that the immune responses of women tend to be stronger, which means that a woman’s lung cancer requires more effective mechanisms to escape the immune system and is therefore less immunogenic than a comparable tumor in a male patient and therefore more resistant to immunotherapy [26, 77]. This is consistent with the observation that men have more partially exhausted cytotoxic T-lymphocytes (peCTLs), such as tumor-infiltrating CD8+ T-cells, which exhibit high amounts of CTLA-4 and PD-1 [78].. These peCTLs seem to be significantly correlated with a better response to anti–PD-1 monotherapy. The observation that additional chemotherapy increases mutational burden and leads to increased non-antigenic exposure of the immune system could explain the greater benefit of this combined treatment strategy in women [27, 79, 80].

#### Eastern Cooperative Oncology Group Performance Status (ECOG-PS)

Various studies on ICI therapy have demonstrated that patients with an excellent or good performance status (ECOG-PS ≤ 2) have a better PFS and OS [28, 29, 81, 82]. Naqash et al. explained this finding with the limited physiological reserves of patients with bad PS which may be a result of either the harmful character and advanced stage of their tumors or from their medical comorbidities [29] . These reduced physiological reserves in turn impair these patients’ tolerance of therapy and therapy-related side effects, causing therapy interruptions or discontinuations. Furthermore, a reduced PS could be an indicator of aggressive tumor biology with intrinsic resistance to ICI therapy. In a meta-analysis of real world NSCLC data including 19 studies it could be shown that PS ≥2 is a poor prognostic factor in terms of RR, PFS and OS for patients treated with ICI [30]. The authors stated that ECOG PS ≥2 patients constitute an inhomogeneous group of patients where the poor clinical condition might be linked with comorbidities, cancer or both. Therefore, patients with an ECOG PS of ≥2 are often excluded in Phase-II and -III trials, nevertheless these patients often show up in the daily clinical practice and need effective treatment options.

#### Body mass index and weight change

In contrast to cardiovascular risk which increases with body weight, it might come as a surprise that patients who presented with a BMI ≥ 25 showed a longer TTF, PFS and OS with ICI treatment than those with a lower BMI [31]. Shepshelovich et al. also reported a U-shaped association between BMI at diagnosis and OS with greater mortality in the extreme groups of underweight and morbidly obese patients, but with the best outcomes in those who are overweight or obese [32]. The results were consistent in all documented tumor entities, disease stages and treatment methods, supposing that this might be a prognostic factor. They did not only compare the BMI at the start of treatment, but also the BMI during youth of the patients, taking a time interval between 18 and 25 years. Relative to the BMI during early adulthood, a decrease in BMI at diagnosis was associated with worse OS when compared to patients who had similar or increased BMI at the time of diagnosis.

Another study analyzed the role of weight change between initial diagnosis and the start of second-line nivolumab immunotherapy in lung cancer patients [33]. They demonstrated that patients with weight loss of more than 5% had a worse OS (median OS of 2 months) than patients who lost less than 5% or even increased their weight (median OS of 10 months). In addition, their results suggest that CT-derived measurements of muscle and fat mass are less clinically relevant than simple, medical history-based assessment of weight loss.

The explanation for this phenomenon might be found in the role of white adipose tissue, which is the effectively increased in obese individuals [31, 83]. This tissue is involved in the induction and coordination of host defenses, being a source of cytokines and chemokines [31, 84]. In fact, adipose tissue modulates the Th1/Th2 balance, decreases the activation of Treg cells through adiponectin and increases pro-inflammatory macrophages as well as the inflammatory status through the CD-40 pathway [31, 85–87]. Individuals with this type of inflammatory status may be more sensitive to the immune checkpoint blockade by PD-1/PD-L1 inhibitors.

### Metastases and their localization

#### Malignant pleural effusion

Several studies have demonstrated, that not only their incidence, but also the localization of metastases is associated with the individual’s prognosis [28, 34]. In particular, the occurrence of malignant pleural effusion seems to be an independent negative predictor for PFS in patients treated with first-line pembrolizumab or second-line nivolumab, possibly because of its correlation with tumor induced immunosuppression [28, 35]. Kawachi et al. hypothesized that this is mediated by vascular endothelial growth factor (VEGF), which promotes malignant pleural effusion by increasing vascular and mesothelial permeability, as well as capillary fluid leakage. Elevated VEGF in patients with malignant pleural effusions is a strong predictor of unfavorable response to pembrolizumab monotherapy in NSCLC patients despite high PD-L1 expression [28, 88–92]. Therefore, the combination of chemotherapy, an ICI agent and bevacizumab, an anti-VEGF antibody, seems to be a promising treatment option in these cases [8, 9, 12, 13, 28, 92–94]. Another problem associated with malignant pleural effusions is the increased number of severe adverse events seen during PD-1 inhibitor based therapy in these patients [34]. This might be explained by higher PD-1 expression on lymphocytes found in the malignant effusions, that may induce excessive immune reactions, which might boost pleural effusion as well as increase the risk of severe advance effects of ICI [34, 95].

### Liver metastases

Furthermore, the presence of liver metastases in NSCLC patients is associated with distinctly worse outcomes irrespective of PD-L1 status [35, 37]. Many authors tried to explain this finding as to why liver metastases represent such a challenge for clinicians. In melanoma patients it has been observed that the density of CD8-positive T-cells at the tumor margin of liver metastasis was markedly reduced and hypothesized that this might reduce the efficacy of ICI therapy [96].

In turn, *Reck* et al. hypothesized that the similarity of NSCLC liver metastasis to hepatocellular carcinoma (HCC) might explain why the combination therapy of atezolizumab + bevacizumab + carboplatin and paclitaxel (ABCP) showed superiority in terms of PFS and OS compared with atezolizumab and chemotherapy (ACP) despite lower PD-L1 expression in patients with liver metastases in the IMpower150 trial [10].

HCC is associated with hypoxic tumor conditions, high VEGF expression and increased angiogenesis, which can contribute to the induction of immunosuppressive cells like regulatory T cells and the promotion of immune tolerance in the tumor microenvironment (TME) [36, 97, 98]. The poor response of patients with liver metastases to ICI monotherapy might be due to this liver-tissue-specific immunoregulation and the addition of bevacizumab potentially reverses this phenomenon [10]. Data seem to be very robust for this ICI and anti-angiogenic combination as the presence of liver metastases was a stratification factor in this trial.

### Brain metastases

Another organ frequently affected by metastases in NSCLC is the brain. The blood-brain barrier reduces the intracerebral availability of drugs and circulating antibodies and prevents the influx of lymphocytes [74]. However, the KEYNOTE-024 trail (first line pembrolizumab) and the KEYNOTE-189 trial (pembrolizumab + pemetrexed & platinum) showed superior OS in all subgroups, even in the group of patients with brain metastasis [8, 17]. This makes ICI therapy a therapeutic option with the need of future prospective trials in patients with active brain metastasis. In a recently published Phase II study in NSCLC patients with CNS metastases without cranial radiotherapy, pembrolizumab monotherapy resulted in an intracranial response in 29.7% of patients with greater than 1% PD-L1 expression. In contrast, no patient with PD-L1 status < 1% had any such response [99].

#### Baseline systemic corticosteroid use

Many NSCLC patients are active or former smokers and thus developed chronic obstructive pulmonary disease (COPD) as a comorbidity. Some of these patients need oral corticosteroids for acute exacerbations or other reasons. However, use of systemic corticosteroids may reduce the clinical beneficial effects of ICIs. In the study of Scott et al. the median OS of patients receiving corticosteroids (≥ 10 mg of prednisone equivalent) during the first 30 days of nivolumab therapy was significantly reduced [38]. This finding was supported by recent retrospective studies [35, 39, 40, 100] and was recently confirmed by retrospective analyses on melanoma, RCC and NSCLC patients treated with nivolumab and pembrolizumab [101]. Patients with a steroid dose of > 10 mg prednisone for more than 2 weeks during ICI treatment had worse outcomes. As a consequence, chemotherapy might be the preferred treatment option, if systemic corticosteroids cannot be avoided in individual patients.

#### Antibiotics given before and during ICI therapy

Patients with NSCLCs are prone to bronchopulmonary bacterial infections and antibiotic therapy may become necessary before or during ICI therapy. However, recent studies show that the antibiotic therapy may be associated with reduced therapeutic effects of ICI therapy. Routy et al. published data suggesting that antibiotic use within 60 days before treatment initiation is associated with a negative effect on OS in a mixed cohort of NSCLC, urothelial carcinoma and RCC patients [41, 102].

In a follow-up study with a larger population of NSCLC patients, it was observed that antibiotic use within 1 month before immunotherapy was associated with a significantly worse OS [42, 102]. These findings are consistent with other results demonstrating that broad spectrum antibiotic therapy within 2 weeks prior to or after ICI use is associated with shorter PFS and a lower response rate [43, 102].

Recently, a pooled post hoc analysis of the OAK and POPLAR trials was published, where atezolizumab was compared with docetaxel in NSCLC patients [44]. 22.3% of patients in the atezolizumab group receiving antibiotics had a significant shorter survival (8.5 months) compared to those ICI patients without anti-microbiologic agents (14.1 months) (HR 1.32, *p* = 0.001). In addition, there was no association between antibiotic use and PFS or OS within the docetaxel population, indicating that antibiotic use was a predictive factor in this analysis. The precise mechanism of these findings has not yet been established. Some experts speculate that antibiotic-related dysmicrobiosis might lead to reduced diversity of the intestinal microbiome and probably eradication of immunogenic bacteria required to support the immune system during ICI therapy [42]. In this context Hakozaki et al. performed a prospective fecal sampling study in 70 NSCLC patients treated with ICI monotherapy [45]. They could demonstrate that those who received antibiotic therapy prior to treatment (22.8%) tended to have a lower bacterial diversity with an underrepresentation especially of *Ruminococcaceae UCG 13* and *Agathobacter* in their intestinal microbiome. These findings were associated with a worse outcome in terms of OS compared to antibiotic-free patients. This suggests that the intestinal microbiome could possibly be developed as a potential predictive marker in the future.

Interestingly, the role of the intestinal microbiome can not only be analyzed by elaborate microbiological examinations, but can also be visualized very elegantly using PET-CT. Cvetkovic et al. analyzed the physiologic colonic uptake of 18F-FDG on PET/CTs of 71 NSCLC prior to ICI therapy [103]. The cohort with a high colonic uptake had a higher risk to experience non-response to ICI and had lowered PFS as well as decreased baseline gut microbiome diversity. Patients were also analyzed according to their cecum uptake, which was also associated with decreased OS in those with a higher cecum uptake. The physiological colonic and cecum uptakes could serve as surrogate markers of gut microbiome diversity, predict clinical outcomes and support the previously mentioned dysbiosis-theory.

### Blood based biomarkers

Blood parameters measured before and during ICI therapy are an important source of information and may help clinicians to decide which therapy to choose and whether ICI might be combined with chemotherapy or anti-angiogenetic agents. Laboratory parameters obtained from blood samples are an easily accessible, non-invasive alternative for parameters analyzed in tissue samples.

#### Baseline Neutrophil-to-Lymphocyte Ratio (NLR) and combination-indexes

Pretreatment elevated baseline NLR calculated from complete blood counts was found to be a prognostic biomarker for patients with NSCLC. A high NLR was a predictor of rapid progression, poor OS and short PFS not only in patients on chemotherapy and targeted therapy, but also in ICI treatment and chemo-ICI combinations [46–48, 51, 82, 104, 105]. Cao et al. analyzed 1225 patients in a meta-analysis and found 1.44-fold better PFS and a 1.75 times higher OS for patients with low pre-treatment NLR (< 5) [46]. In contrast low NLR and PLR (platelet-to lymphocyte ratio) at baseline were reported to be significantly associated with the development of immune related toxicities in NSCLC patients [82]. However, only PLR was an independent prognostic factor for toxicity in this retrospective analysis.

In immunotherapy it is hypothesized that, due to the systemic inflammation initiated by the tumor itself, the increased infiltration of neutrophils promotes cancer progression via secreting interleukin-10 (IL-10), tumor necrosis factor α (TNF-α), and VEGF [104, 106]. TNF-α and IL-10 both reduce the number of lymphocytes and inhibit with T-lymphocyte-mediated antitumor effects. Accordingly, the NLR has the potential to serve as a predictive marker for the outcome of ICI therapy [104]. NLR is also used as part of various indexes like Systemic-Immune-Inflammation Index (SII) [platelet count x NLR] [49, 107]. Here Liu et al. established the SII of 44 NSCLC patients before 2nd line nivolumab treatment initiation and reported an increased OS and PFS for patients with a low SII (< 603.5) [median OS: 19.8 months; median PFS: 6.9 months]. The Lung Immune Prognostic Index (LIPI) used by Ruiz-Banobre et al. combines NLR > 3 and LDH greater than upper limit of norm [108]. They classified their cohort of 153 NSCLC patients receiving 2nd line nivolumab in three groups according to LIPI and reported median OS for their poor, intermediate, and good LIPI subgroups to be 3.4 months, 7.3 months and 20.8 months respectively [108].

#### Lactate Dehydrogenase levels (LDH)

In a meta-analysis out of six studies with 1136 patients, Zhang et al. confirmed the hypothesis that low pretherapeutic LDH levels were correlated with significantly longer PFS and OS of NSCLC patients treated by ICI [53]. LDH levels seem to be related to intratumoral hypoxia and increased angiogenesis mediated by macrophages [109–113]. However, it remains uncertain which cut-off value of LDH is best to estimate the survival of patients with NSCLC. The values varied in the individual studies between 217 and 400 U/L [53]. Furthermore, since LDH is a dynamic marker, the optimal time and frequency of LDH measurements is also unclear [53]. It was hypothesized that only decreasing LDH levels from baseline to week 12 of CTLA4-antibody therapy were associated with longer PFS and OS in melanoma patients [114, 115]. However, these data need further confirmation.

#### C-Reactive Protein (CRP)

C-reactive protein (CRP) is an acute phase protein of hepatic origin that reflects tissue injury and systemic inflammation. Since inflammation forms an essential aspect of the neoplastic process, it came as no surprise that elevated baseline CRP levels seems to be a predictive marker for better PFS and OS after ICI initiation in NSCLC and melanoma patients [35, 48, 54, 116, 117]. We recently could show that a doubling of baseline CRP level was associated with a 1.4-fold higher relative risk of disease progression or death and that a 10 mg/mL/month faster increase in CRP levels over time predicted for 13-fold higher risk of experiencing a PFS event. In addition, a decreasing CRP during the first 8 weeks after ICI initiation was strongly associated with favorable PFS experience (10% reduction in CRP predicted for a 0.9-fold reduction in PFS) [118].

A locally inflamed TME promotes cancer progression by stimulating cell proliferation, angiogenesis and cancer cell migration. Furthermore, cancer cells also promote systemic inflammation, which is induced by diverse immune cells, cytokines and acute phase proteins [119, 120]. Systemic inflammation often indicates cancer progression and is considered to cause cancer-related complications such as cachexia, pyrexia and fatigue [119, 121–127]. Moreover, systemic inflammation as reflected by serum CRP has been suggested to correlate with low levels of CD4+ T-cells, which play a key role in ICI mediated antitumor immune response [128].

### Genetic markers from tumor tissue and/or blood

#### Soluble PD-L1 in plasma

Beside the immunohistochemical PD-L1 staining on tumor cells, which is currently a standard procedure in the initial histological diagnosis of NSCLC, plasma markers are also of particular importance. Two forms of PD-L1 are evident: a membrane-bound one (mPD-L1) and a soluble form (sPD-L1) that may be present in plasma of patients with solid tumors [129]. The key advantage of using sPD-L1 as a clinical marker is its easy accessibility without the need to obtain additional tumor tissue thus giving the opportunity of easy repetitive measurements during ICI therapy. Okuma et al. analyzed the plasma of stage IV NSCLC patients treated with nivolumab in 2nd -line setting and measured baseline sPD-L1 with ELISA. Patients with high sPD-L1 levels (cutoff value: 3357 ng/mL) demonstrated significantly shorter OS and a reduced time to treatment failure (TTF). Conversely, low levels of sPD-L1 were associated with high rates of tumor control [55, 129]. In another study sPD-L1 levels were measured at initial diagnosis, at nivolumab initiation and at first post-therapeutic tumor evaluation in NSCLC patients [56]. It was observed that increasing sPD-L1 levels between the initiation of ICI therapy and first tumor evaluation were associated with worse ORR, shorter PFS and reduced OS. Higher sPD-L1 levels were found in non-responders and low levels were associated with clinical benefits [56]. A major limitation for the clinical implementation of sPD-L1 as a prognostic marker during routine care is the lack of standardization of sPD-L1 measurements, with a number of different ELISA kits and varying thresholds [129].

#### VEGF in serum

Vascular endothelial growth factor (VEGF) plays an essential role in tumor angiogenesis, which is crucial for cancer proliferation, migration and metastasis [57]. In addition, inhibition of dendritic cell maturation, reduction of T-cell tumor infiltration, and promotion of inhibitory cells in the tumor microenvironment (TME) appear to be important in VEGF-mediated immunosuppression [130, 131]. Antiangiogenic agents could not only antagonize these VEGF-driven effects, but could also affect tumor blood vessels by reducing their size and length in the tumor and promoting vessel maturation, resulting in improved tissue penetration of chemotherapy and ICI substances [132–134].. We have recently demonstrated the effectiveness of this in NSCLC patients [135]. NSCLC patients receiving docetaxel and ramucirumab, a VEGFR antibody, in the 3rd line after 2nd line treatment with an ICI not only had a particular encouraging mOS of nearly 1 year, but also showed an increased response rate of this combination compared to the previously given immunotherapy.

VEGF expression can be easily measured by ELISA and seems to be negatively associated with survival in NSCLC patients when measured in the tumor [136–138]. Furthermore, high pretreatment serum levels of VEGF have been shown to have a prognostically negative value in patients undergoing palliative chemotherapy with gemcitabine and vinorelbine [139]. Surprisingly, there is little data on VEGF in pretreatment tissue from NSCLC patients receiving ICI. In a small retrospective cohort of NSCLC patients receiving PD-1 treatment, high levels of pretreatment serum VEGF were a significant predictor of shorter progression free survival, especially in elderly (> 75 years) and ECOG PS 2 patients [57]. Further analyses of this promising marker are necessary to assess its further prognostic and/or predictive potential.

#### Tumor Mutational Burden (TMB)

A common hypothesis among experts is that tumors with a high mutational burden express also many neoantigens, which are captured and presented by dendritic cells and thus leading to a high diversity of primed T-cells [140, 141]. The more diverse T-cells are, the higher is their chance of localizing and destroying tumor cells. One particular impressive example are patients presenting with a DNA mismatch repair mutation. In these patients many different proteins and epitopes are generated through microsatellite instability. Excellent results with ICI treatment in those patients led to approval across a whole variety of tumor types [142–144]. However, there is no consensus among experts as to which mutations should be used for the calculation of tissue Tumor Mutational Burden (tTMB). Some authors report all mutations, others use only non-synonymous mutations, and yet others consider only missense mutations in exons as relevant [145–151].

At last year’s ESMO congress Herbst et al. presented exploratory data from the KEYNOTE-010 and KEYNOTE-042 trials, showing that a high tTMB ≥175 mutations per exome might be of positive predictive value [152]. Patients in these studies treated with pembrolizumab monotherapy with a high tTMB level had a significant better outcome in terms of ORR, PFS and OS in contrast to patients treated with chemotherapy. Conversely, tTMB was not associated with outcomes in the chemotherapy-arm in either study. In contrast, no association between tTMB levels and efficacy in neither RR, PFS nor OS of pembrolizumab and/or chemotherapy were found in the KEYNOTE-021, − 189 or 407 studies [60]. Therefore, tTMB is not established as a marker for ICI efficacy in NSCLC so far.

Less invasive methods have been sought, such as measuring TMB on circulating tumor DNA (ctDNA) in patient plasma termed blood TMB (bTMB). Wang et al. evaluated bTMB with the cancer gene panel NCC-GP150, which was designed and virtually validated using the Cancer Genome Atlas database [129, 153]. The correlation between ICI-treated patients’ bTMB estimated by NCC-GP150 and tissue TMB (tTMB) measured by whole exome sequencing (WES) was evaluated in matched blood and tissue samples from 48 patients with advanced NSCLC and a bTMB ≥6 was associated with an increased PFS, clinical tumor shrinkage and superior ORR. This way of measuring needed a smaller panel size (only 150 genes) and showed superior cost effectiveness compared to tTMB evaluation. However, this approach has to be tested in prospective trials before being used in the clinical setting.

Taking the KEYNOTE-189 trial again, 235 participants’ bTMBs have been analyzed post hoc and the results were presented recently [154]. Garassino et al. observed that a bTMB > 15 mut/mB was associated with worse PFS in the pembrolizumab + chemotherapy-arm, but not with reduced OS and that tTMB was not significantly associated with efficacy in both groups, what confirmed the previously mentioned results. Nevertheless, Rizvi et al. recently observed improvements in OS for patients treated with durvalumab (D) + tremelimumab (T) with a bTMB ≥20 mut/mB in their exploratory phase-III trial (MYSTIC) where first-line D + T was compared with chemotherapy (21.9 months vs 10.0 months) [59]. This study did not meet its primary endpoints but is still highlighting the need for further investigation and prospective validation of blood tumor mutational burden as a predictive biomarker for immunotherapy agents.

### Driver mutations and translocations

The identification of somatic mutations and translocations in adenocarcinoma of the lung is common practice and allows targeted therapy with tyrosine kinase inhibitors (TKI), especially for patients with stage IV NSCLC. Interestingly enough, a retrospective study showed that most patients with a targetable driver mutation/translocation hardly have any benefit from ICI therapy [155, 156].

In most phase III trials patients with known driver mutations/transloctions, especially EGFR and ALK were excluded. The only trial were those patients were treated was the ImPower 150 study [21]. It could be shown, that those patients though not stratified had a significant benefit in terms of OS from a chemo-antiangiogenesis-ICI combination and that especially the ICI is relevant for the outcome. Further trials should include patients with driver mutations at least after progression to a TKI.

In western countries, the most frequent driver mutation in adenocarcinoma is found in the Kirsten rat sarcoma viral oncogene homolog (KRAS) [157–159]. RAS proteins act as a cellular switch that is turned on by extracellular stimuli activating different signaling pathways that regulate fundamental cell processes [158, 160]. Activating KRAS mutations in adenocarcinoma are usually found in smokers and unlike other oncogene-driven lung cancers, they frequently appear with other major genetic co-mutations [158, 159]. In two recently reported exploratory analyses of trials where pembrolizumab was used either as monotherapy (KN042) or in combination with chemotherapy (KN189), ICI was superior to chemotherapy alone irrespective of the KRAS status [61, 62]. However, while there was no difference among patients in the ICI-chemotherapy arm as a consequence of the KRAS status, an improved ORR and a longer PFS time were seen in the ICI monotherapy arm when the tumors exhibited a KRAS mutation. Furthermore, in the pembrolizumab mono therapy arm, there was a benefit in OS of those patients exhibiting a KRAS mutation of 28 vs 15 months for those with the wild-type.

### STK11/KEAP1

Many previous published studies examined the role of co-mutations in ICI treated driver mutation-positive adenocarcinoma. Tumor-cell-intrinsic oncogenic pathways including STK11/LKB1 and KEAP1 are associated with a “cold”, non-T cell-inflamed TME, thus significantly impairing clinical responses to ICI treatment [22, 63, 64, 161, 162]. The inactivation of STK 11 increases production of G-CSF, CXCL7 and IL-6, which recruit tumor-associated neutrophils that in turn increase T-cell dysfunction via arginase A and IL-10 release [22, 162]. Along with that, this genomic alteration has been identified as a major driver of de-novo resistance to PD-1 axis resistance in KRAS-positive lung adenocarcinoma [63].

Linked with the inactivation of STK11 is the loss of KEAP1, an adaptor protein that mediates ubiquitination and proteasomal degradation of NRF2, which in turn is a key transcription factor in cellular antioxidant, metabolic, cytoprotective and anti-inflammatory pathway [162, 163].

At this year’s ASCO congress, Pavan et al. presented their results about the impact of STK11, KRAS and TP53 mutations on the clinical outcome of prospectively screened NSCLC patients treated with ICI therapy in the VISION and MAGICAL trail. They detected that STK11 mutated patients have a trend for worse OS compared with wild-type counterpart in patients undergoing immunotherapy. This phenomenon was not seen in the control group receiving no ICI medication, making STK11 a possible negative predictive factor for ICI therapy. In contrast, Cho et al. made a post hoc analysis of the KEYNOTE-042 study and observed that pembrolizumab monotherapy had similar efficacy in patients regardless of STK11 or KEAP1 status, but an increased efficacy compared to the platinum-based chemotherapy arm [65]. Since they also detected a lower PD-L1 expression in the STK11 mutated group, there is a need for more prospective studies to evaluate this approach.

### TP53

Since TP53 tended to be associated with an Inflamed or “hot” tumor environment, some experts stated that patients with this mutation should have a high rate of clinical response to PD-1 axis immunotherapy, increased PD-1 expression and an increased PFS and OS in a NSCLC trial [162]. They assumed that the biological background might be the activation of NF-ΚB through the loss of TP53, which increases production of inflammatory cytokines [22, 161]. However, Pavan’s et al. analysis identified TP53 as a negative prognostic marker which was associated with decreased OS in both, the ICI cohort as well as the control group (95% CI: 1,2-5,2; *p* = 0,014). As the results regarding the impact of TP53 mutations have currently such a large variation, there is an urgent need for larger, systematic studies addressing this question.

#### Circulating tumor DNA (ctDNA)

Circulating tumor DNA (cTDNA) and RNA (cTRNA), shed by tumor cells into the blood can be used as a liquid biopsy, providing noninvasive access to cancer-specific mutations [164]. In particular, the detection of EGFR sensitizing mutations before starting therapy and the detection of acquired resistance mutations during therapy with EGFR tyrosine kinase inhibitors (TKIs) has become a standard clinical method in recent years [165]. In addition, in patients receiving 1st-line TKI treatment, ctDNA follow-up measurements of activating EGFR mutations can be used to monitor the effectiveness of treatment. Patients treated with osimertinib in the Phase III FLAURA study, in whom the mutation is no longer detectable 3 or 6 weeks after the start of the treatment, had significantly higher PFS (HR 0.57 and 0.51) compared to patients in whom the mutation was retained [166].

Longitudinal ctDNA analyses are also in the focus of translational research during ICI therapy. In the recently published INSPIRA trial, a prospective phase II study, 94 patients with solid tumors treated with pembrolizumab where included and serial plasma samples were obtained before and during therapy [66]. Baseline ctDNA concentrations below the mean were correlated with an increased response rate and better outcome in terms of PFS and OS. Furthermore, a reduction or degradation of ctDNA during ICI therapy indicated a favorable risk group. In contrast, a dynamic increase in ctDNA over time was associated with an early progress of the disease. In line with these results are the data presented by Bonanno et al. at the recent 2020 ESMO congress on longitudinal ctDNA analyses of plasma samples from NSCLC patients treated by ICI [167]. An increase of the mean fold change in the variant allele fraction between start and after 3–4 weeks of treatment was observed frequently in patients with an early progress or even a hyperprogression. The same research group followed 34 NSCLC patients under ICI treatment with longitudinal ctDNA analysis and detection of KRAS mutations in the blood [67]. The detection of a KRAS mutation before treatment had no clear prognostic value. However, a new KRAS mutation becoming apparent after 3 to 4 weeks of therapy was associated with a significant shorter PFS and a trend towards a shorter OS.

In summary, ctDNA monitoring is not yet ready for clinical practice because the cohorts studied so far are still small and different molecular methods make them difficult to compare. So far, however, all published results go in the same direction and show that a decrease in ctDNA during treatment is associated with a favorable outcome, while an increase is often associated with an early progression or an even hyperprogressive disease. Close ctDNA monitoring could become a valuable tool for therapy guidance in the near future.

## Conclusion

Immunotherapy is a new and exciting treatment approach in NSCLC patients. The pivotal challenge will be to guide the choice of individual therapeutic concepts and how to target the TME and tumor itself appropriately at the right time. There is a great number of clinical-, laboratory- and genetic data, that might help to predict the individual response to ICI therapy, but so far, these data are not incorporated comprehensively into clinical studies and most of these assumed predictive factors are not ready for daily routine yet. While the understanding of the mode of action of ICI therapy and their interaction with the immune response as well as TME grows further, we probably will discover more and more relevant genetic parameters which might be helpful for clinical decision making. The performance of predictive biomarkers for ICIs might also be improved by combining different markers to improve their specificity. Many clinical studies and a significant amount of translational research on easily accessible, cost-effective, standardized biomarkers and measurement techniques is still needed to develop reliable algorithms for treatment choice for patients with NSCLC.

In summary Fig. 1 gives a comprehensive overview about the clinically relevant positive prognostic and/or predictive factors.

## Data Availability

All data have been either published either as full text in PubMed or were presented at one of the recent international oncology symposia. Where full presentations of studies or congress data cannot be obtained, the datasets used are available from the corresponding author on request. Figure 1 was designed using free vector icons from Graphic Surf (https://graphicsurf.com).

## Abbreviations

BMI: Body mass index
bTMB: Blood tumor mutational burden
CI: Confidence interval
COPD: Chronic obstructive pulmonary disease
CR: Complete response
CRP: C-reactive protein
ctDNA: Circulating tumor DNA
ctRNA: Circulating tumor RNA
CTC: Common toxicity criteria
CT: Computer tomography
DCR: Disease control rate
DOR: Duration of response
ECOG PS: Eastern Cooperative Oncology Group performance status
EGFR: Epidermal growth factor receptor
FDA: Food and Drug Administration (USA)
HCC: Hepatocellular carcinoma
HR: Hazard ratio
ICI: Immune-checkpoint inhibitor
KRAS: Kirsten rat sarcoma viral oncogene homolog
LDH: Lactate dehydrogenase
LIPI: Lung Immune Prognostic Index
NGS: Next generation sequencing
NSCLC: Non-small cell lung cancer
PD: Progressive disease
RECIST: Response Evaluation Criteria in Solid Tumors version 1.1.8
PD-1: Programmed cell death protein 1
PD-L1: Programmed cell death-ligand 1
peCTL: Partially exhausted cytotoxic T-lymphocytes
ORR: Objective response rate
OS: Overall survival
PFS: Progression-free survival
PR: Partial response
RCC: Renal cell carcinoma
SD: Stable disease
SII: Systemic-Immune-Inflammation Index
sPD-L1: Soluble programmed cell death-ligand 1
TKI: Tyrosine kinase inhibitor
TME: Tumor microenvironment
TNF-α: Tumor necrosis factor α
TPS score: Tumor proportion score
Treg: Regulatory T cell
TTF: Time to treatment failure
tTMB: Tissue tumor mutational burden
UICC: Union for International Cancer Control
VEGF: Vascular endothelial growth factor
VEGFR: VEGF receptor
